# Lupus Enteritis: An Unusual Flare of Systemic Lupus Erythematosus

**DOI:** 10.7759/cureus.99784

**Published:** 2025-12-21

**Authors:** João Casanova Pinto, Manuel G. Costa, Beatriz Fernandes, Carlos Ramalheira

**Affiliations:** 1 Internal Medicine, Hospital de Cascais, Dr. José de Almeida, Lisbon, PRT; 2 Facultat de Medicina i Ciències de la Salut, Universitat de Barcelona, Barcelona, ESP; 3 NOVA Medical School, Universidade Nova de Lisboa, Lisbon, PRT

**Keywords:** chronic diarrhea, lupus enteritis, lupus mesenteric vasculitis, mesenteric vasculitis, systemic lupus erythromatosus

## Abstract

A woman in her 20s with previously diagnosed systemic lupus erythematosus (SLE) presented with a year-long history of chronic watery diarrhea, significant weight loss, and additive symmetrical inflammatory polyarthritis. She had been receiving hydroxychloroquine, azathioprine, and low-dose prednisolone but had discontinued treatment shortly before admission due to persistent vomiting. Laboratory evaluation showed new-onset proteinuria, hypokalemia, hypomagnesemia, microcytic anemia, lymphopenia, low complement levels, and markedly elevated antinuclear and anti-double-stranded deoxyribonucleic acid (anti-dsDNA) antibodies. Imaging demonstrated diffuse mural thickening and submucosal edema of the small bowel, a large left pleural effusion, and peritoneal fluid. The patient was treated with intravenous methylprednisolone pulses, followed by high-dose oral prednisolone and hydroxychloroquine. Renal biopsy revealed class III lupus nephritis, leading to the initiation of mycophenolate mofetil (MMF). Under this immunosuppressive regimen, gastrointestinal symptoms resolved, pleural effusion regressed, and renal parameters progressively improved.

This case illustrates lupus enteritis as a rare but clinically significant manifestation of SLE, presenting with chronic diarrhea in association with other systemic features, including inflammatory polyarthritis, hematological involvement, and lupus nephritis. This report emphasizes the importance of considering lupus enteritis in patients with SLE who present with persistent gastrointestinal symptoms and of early recognition and prompt initiation of appropriate immunosuppressive therapy to prevent severe complications such as ischemia and perforation.

## Introduction

Systemic lupus erythematosus (SLE) is an autoimmune connective tissue disease characterized by diverse clinical features and multi-organ involvement. While it often affects the musculoskeletal, renal, and hematologic systems, gastrointestinal manifestations can also occur. Among these, lupus enteritis (LE) is relatively rare but possibly severe. It is defined as the presence of bowel-wall inflammation in patients with SLE activity, and it is usually present in the context of multiorgan involvement [1]. Its presentation can overlap with common conditions such as infectious gastroenteritis or medication-induced enteropathy, thus complicating early diagnosis.

In LE, patients may exhibit nonspecific findings, including diarrhea, abdominal pain, vomiting, or malabsorption [2,3]. Diagnostic challenges often stem from concurrent disease flares in other systems and a broad differential diagnosis for gastrointestinal complaints. Failure to recognize LE can lead to complications such as intestinal ischemia and perforation [4]. This case report discusses an unusual lupus flare with prominent enteric involvement, highlighting the necessity for heightened clinical suspicion and rapid intervention to prevent morbidity.

This case was presented as an abstract/poster at the 22nd European Congress of Internal Medicine in 2024.

## Case presentation

Approximately one year prior to the current admission, a previously healthy woman in her 20s developed chronic diarrhea with three to four daily liquid bowel movements, without blood, mucus, tenesmus, or greasy/yellowish appearance. Over the following months, this was accompanied by anorexia and marked unintentional weight loss from 80 kg to 55 kg (31.25% of total body weight). She subsequently developed fatigue and symmetrical inflammatory polyarthritis involving the wrists, hands, and knees, with associated morning stiffness lasting longer than one hour. She denied other systemic features including photosensitivity, cutaneous changes, oral or nasal ulcers, Raynaud's phenomenon, *sicca* symptoms (xerostomia or xerophthalmia), alopecia, chest pain, neuropsychiatric symptoms, and urinary complaints, including hematuria. Three months prior to admission, in an outpatient setting, she had been diagnosed with SLE based on positive antinuclear antibodies, elevated anti-double-stranded deoxyribonucleic acid (anti-dsDNA) titers, and clinical features that included arthritis, hematological involvement, and significant weight loss. She was started on hydroxychloroquine (HCQ) 400 mg once daily, azathioprine 75 mg once daily, and prednisolone 5 mg once daily. Two weeks before the hospitalization, she stopped taking her medications due to persistent vomiting. Following the discontinuation of therapy, her symptoms worsened, and she presented to the emergency room with persistent watery diarrhea (more than six liquid stools per day), ongoing polyarthritis, and generalized fatigue.

On admission, the patient appeared fatigued, and signs of dehydration stood out. Regarding her vital signs, she was afebrile (35.6 ºC), presenting mild sinus tachycardia (105 bpm) and borderline low blood pressure (98/64 mmHg). Examination of the chest revealed diminished breath sounds over the lower third of the left hemithorax. Abdominal palpation elicited periumbilical tenderness without overt guarding or rebound. Examination of her joints confirmed signs of active arthritis in both wrists and knees. Initial laboratory results demonstrated microcytic anemia, mild leukopenia (due to moderate lymphopenia), normal platelets, hypokalemia, and hypomagnesemia. Other ions were normal. The iron panel showed no alterations, and both vitamins B9 and B12 were normal. Renal function was reported as normal, yet urinalysis revealed an active urinary sediment characterized by glomerular hematuria with dysmorphic erythrocytes, numerous red blood cell casts, sterile leukocyturia, and granular casts, associated with sub-nephrotic-range proteinuria. Complement levels were low. The autoantibody panel revealed high-titer antinuclear antibodies, elevated anti-dsDNA, positive anti-single-stranded deoxyribonucleic acid (anti-ssDNA), anti-histones, anti-Ro/SSA, anti-La/SSB, and anti-ribonucleoprotein (anti-RNP), while anti-Smith (anti-Sm) antibody, rheumatoid factor, and anti-neutrophil cytoplasm antibodies (ANCA) were negative (Table 1).

**Table 1:** Blood test results on admission ANA: antinuclear antibody; ANCA: antineutrophil cytoplasmic antibody; anti-dsDNA: antibody against double-stranded deoxyribonucleic acid; anti-ssDNA: antibody against single-stranded deoxyribonucleic acid; anti-La/SSB: antibody against La/SSB antigen (Sjogren syndrome type B); anti-Ro/SSA: antibody against Ro/SSA antigen (Sjogren syndrome type A); Anti-RNP: anti-ribonucleoprotein antibody; Anti-Sm: antibody against Smith antigen.

Test	Result	Reference Range
Serum creatinine (mg/dL)	0.52	0.50 – 0.90
Urinary protein to creatinine ratio (mg/g)	1528	< 200
Serum sodium (mmol/L)	136	135 – 145
Serum potassium (mmol/L)	3.3	3.5 – 5.0
Serum magnesium (mg/dL)	1.3	1.6 – 2.4
Serum calcium (mg/dL)	8.7	8.5 – 10.5
Hemoglobin (g/dL)	9.0	12.0 – 15.5
Mean corpuscular volume (fL)	83	80 – 100
Leukocytes (x 10^9^/L)	3,450	4,500 – 11,000
Neutrophils (x 10^9^/L)	2,550	2,500 – 7,500
Lymphocytes (x 10^9^/L)	550	1,000 – 4800
Platelets (x 10^9^/L)	154	150 – 450
Ferritin (ng/mL)	153	13 – 150
Transferrin saturation (%)	22	20 – 50
Erythrocyte sedimentation rate (mm/h)	94	< 20
C-reactive protein (mg/dL)	12.40	< 0.3
C3 complement (mg/dL)	32	84 – 160
C4 complement (mg/dL)	7.5	12.0 – 36.0
Antinuclear antibodies	>1:1280 (Positive) AC-1 pattern	< 1:80 (Negative)
Anti-dsDNA (U/mL)	>379 (Positive)	< 30 (Negative)
Anti-ssDNA (U/mL)	>528 (Positive)	< 20 (Negative)
Anti-Histones	+++ (Positive)	Negative
Anti-Ro/SSA	+++ (Positive)	Negative
Anti-La/SSB	+++ (Positive)	Negative
Anti-RNP/U1 RNP	+++ (Positive)	Negative
Anti-Sm	Negative	Negative
Rheumatoid factor (U/mL)	<10.0	< 14.0
ANCA	Negative	Negative

**Figure 1:**
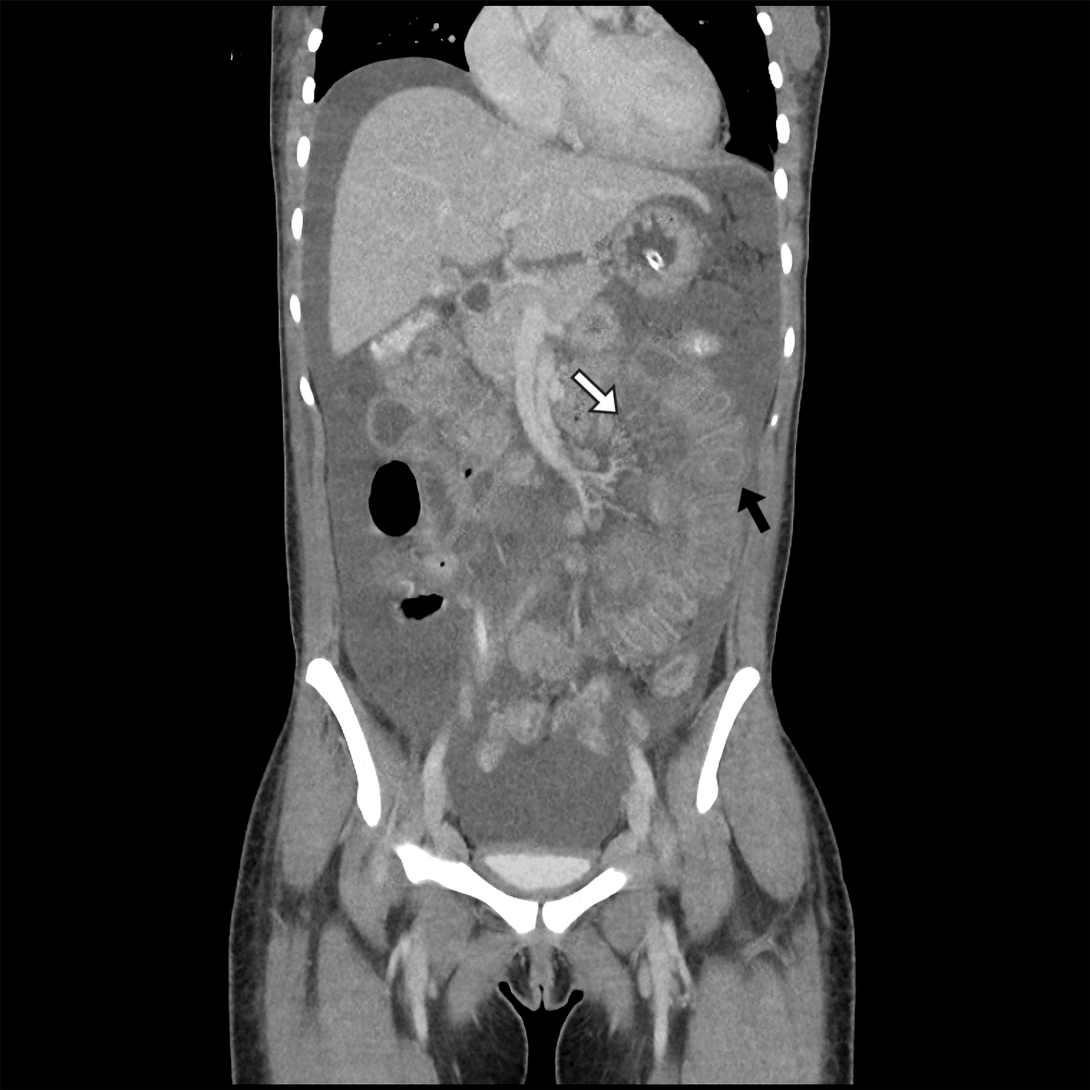
Coronal venous-phase contrast-enhanced abdominopelvic computed tomography (CT) image. This study demonstrates diffuse, circumferential thickening of multiple small-bowel loops, predominantly involving the jejunum and ileum, associated with marked submucosal edema, resulting in a characteristic “target” or “double-halo” appearance of the transversally seen bowel loop (black arrow). These findings are accompanied by mesenteric fat stranding and vessel engorgement, also referred to as a "comb sign" (white arrow). A moderate amount of free intraperitoneal fluid is also noted. As a whole, these findings can be attributable to an inflammatory small bowel process, in this case an active lupus enteritis.

Contrast-enhanced computed tomography (CT) of the thorax, abdomen, and pelvis demonstrated bilateral pleural effusion and moderate ascites. A diffuse and regular small bowel wall thickening was found with maintained mucosal enhancement. It mainly involved the jejunum, where an edematous hypoenhancing submucosal layer was found. These findings are suggestive of an inflammatory or ischemic small bowel process, consistent with LE involvement (Figure 1). Mild retroperitoneal and pelvic lymphadenopathies were present. Transthoracic echocardiogram found no pericardial effusion.

A comprehensive diagnostic evaluation was performed to exclude alternative causes of chronic diarrhea. Repeated stool studies, including bacterial cultures, *Clostridioides difficile* toxin polymerase chain reaction (PCR), ova, and parasite examination, as well as extended molecular testing for enteric pathogens, were negative. Colonoscopy with ileoscopy revealed a nonspecific colitis without distinctive macroscopic features; however, extensive mucosal biopsies excluded inflammatory bowel disease, cytomegalovirus infection, and malignancy. Normal fecal calprotectin, normal fecal elastase, and negative celiac serology with normal total IgA argued against inflammatory bowel disease, pancreatic exocrine insufficiency, and celiac disease, respectively. ANCA testing was negative, and small-vessel vasculitis was therefore considered unlikely. Endocrine causes, including hyperthyroidism, diabetes mellitus, and Addison's disease, were excluded by appropriate laboratory testing. Bile acid malabsorption was deemed unlikely due to the absence of ileal disease or resection. A thorough medication review excluded drug-induced diarrhea. In contrast, positive immunologic markers together with imaging findings of diffuse bowel-wall thickening and edema supported an inflammatory mechanism. On this basis, the most plausible diagnosis was a flare of SLE with gastrointestinal involvement, consistent with LE.

Upon admission, intravenous methylprednisolone was administered at a dose of 1 g daily for three days. Oral prednisolone at 1 mg/kg/day was initiated on day four. HCQ was restarted. Intravenous fluid support and electrolyte replacement were provided to correct volume and electrolyte imbalances. On day six, an ultrasound-guided thoracentesis drained 1300 mL of serous pleural fluid compatible with a transudate, negative for malignant cells and mycobacteria. A renal biopsy performed on day eight confirmed class III lupus nephritis. Consequently, mycophenolate mofetil (MMF) was added at a dose of 500 mg twice daily. She showed progressive improvement of the gastrointestinal symptoms, with reduced diarrhea to one to two watery stools per day, and better oral intake. Fatigue and articular symptoms also ameliorated, with the patient reporting feeling notably better. Pleural effusion diminished, and proteinuria decreased (urinary protein to creatinine ratio of 1135 mg/g). By the 10th day of hospitalization, she was hemodynamically stable, and serum potassium and magnesium had normalized. She was discharged on the 12th day of hospitalization under MMF 500 mg twice daily, prednisolone 20 mg once daily, HCQ 400 mg once daily, ramipril 5 mg once daily, omeprazole 20 mg once daily, and cotrimoxazole 960 mg once daily. She was counseled against pregnancy due to the potential teratogenic effects of MMF and the risk of triggering a lupus flare. Arrangements were made for close outpatient monitoring of renal function, disease activity, and medication tolerance. At one-year follow-up, the patient had achieved disease control, with a Systemic Lupus Erythematosus Disease Activity Index 2000 (SLEDAI-2K) < 4 and a proteinuria < 0.7 g/24h under HCQ 400 mg per day, MMF 1500 mg twice daily, and a prednisolone dose < 5 mg per day.

## Discussion

SLE is a heterogeneous autoimmune disease in which gastrointestinal involvement is reported in approximately 20-40% of patients [5]. LE represents an infrequent manifestation within this spectrum, with a prevalence ranging from 0.2% to 9.7% among SLE cohorts, yet it carries the potential for significant morbidity. The condition predominantly affects women and is characterized by inflammatory and vasculopathic changes of the bowel wall with associated edema, potentially involving any segment of the gastrointestinal tract, although the small intestine is most often affected [1,6].

LE may occur at different points during the course of SLE, most commonly developing three to four years after the initial diagnosis [6]. Two main physiopathological mechanisms have been proposed for LE: an inflammatory mechanism and a thrombotic mechanism. The inflammatory pathway is driven by immune complex deposition and complement activation within the intestinal microvasculature, leading to vascular inflammation, increased permeability, and bowel wall edema [4]. The thrombotic mechanism is related to superimposed vasospasm or *in situ* microvascular thrombosis, frequently associated with antiphospholipid antibodies, which may further compromise intestinal perfusion, resulting in transmural ischemic injury. In severe cases, these processes may culminate in ulceration and intestinal perforation [6].

Clinical presentation is typically nonspecific and most frequently includes abdominal pain (97%), ascites (78%), nausea (49%), vomiting (42%), and diarrhea (32%) [7]. The patient’s pronounced unintentional weight loss of over 30% of her total body weight represents an important marker of disease chronicity and severity. In LE, significant weight loss may result from prolonged malabsorption, chronic intestinal inflammation, reduced oral intake due to persistent gastrointestinal symptoms, and increased systemic inflammatory burden. In the present case, the patient’s prolonged history of diarrhea, anorexia, and weight loss over one year suggests chronic intestinal involvement rather than an acute flare. This indolent course may obscure the diagnosis and contribute to delayed recognition [8,9].

LE frequently coincides with active systemic disease, especially biopsy-proven lupus nephritis and hematological abnormalities, which is mirrored in the presented case [7]. LE also tends to correlate with elevated lupus activity indices. In this case, the patient scored 22 points on the SLEDAI-2K. Nevertheless, it is important to note that standard severity scores may not reflect the severity of SLE in patients who present with LE [6]. LE is commonly associated with hematologic abnormalities, including leukopenia, lymphopenia, and anemia, as well as frequent immunological findings such as ANA positivity (92%), anti-dsDNA antibodies (74%), and hypocomplementemia (88%). Anti-RNP (28%), anti-SSA (26%), and anti-Sm (24%) antibodies are less commonly detected. Notably, C-reactive protein elevation is not a typical feature of this entity [7]. In our case, the patient exhibited all of the typical and some of the atypical laboratory abnormalities previously described in LE, with the sole exception of anti-Sm antibodies, which were negative. The presence of low complement levels, high anti-dsDNA antibodies, and systemic features such as pleural effusions and nephritis pointed to a global flare of SLE rather than an isolated gastrointestinal pathology [10].

Contrast-enhanced CT remains the gold-standard imaging diagnostic tool, often demonstrating the classic “target sign” (concentric bowel wall thickening where the hyperenhancing mucosa contrasts with the hypoenhancing and thickened submucosal layer), as well as the “comb sign” (prominent mesenteric vessels) [6,11]. In this patient, signs of an inflammatory or ischemic small-bowel process were found, aligning with previous reports describing radiologic manifestations of LE [12]. Due to the superficial nature of most gastrointestinal biopsies, histopathological findings in LE are frequently nondiagnostic, with vasculitis or necrosis observed in only a minority of cases [7]. Endoscopy is primarily useful for excluding alternative diagnoses rather than confirming LE. When present, endoscopic abnormalities are typically nonspecific and include mucosal edema, erosions, or ulcerations [7]. Both endoscopic and intestinal histopathological findings in our patient align with these data.

Other etiologies to be considered are GI infections, including opportunistic infections in immunosuppressed patients, other immune-mediated diseases, such as inflammatory bowel disease (IBD) and ANCA-associated vasculitis, and drug-induced toxicity, namely those associated with immunosuppressive agents. These causes were excluded by the combination of clinical, laboratorial, radiological, and histological features previously presented.

Management of LE relies on controlling the underlying SLE flare. High-dose corticosteroids are the mainstay of acute treatment, often leading to rapid improvement of bowel symptoms [13]. In refractory cases or when concomitant organ involvement exists, additional immunosuppressive agents may be employed. The choice most often falls on cyclophosphamide, MMF, or rituximab, the latter being the most effective one in treating unresponsive patients [9,14]. This patient responded to intravenous methylprednisolone followed by oral prednisolone (on a tapering scheme), MMF, and HCQ. The data mentioned above shows that the patient has had sustained clinical remission at one-year follow-up. This case aligns with existing literature indicating that LE can be a hallmark of severe lupus flares, typically responding to aggressive immunosuppression [5]. LE can occasionally relapse, especially in cases with suboptimal adherence to therapy. Long-term monitoring is important, as recurrence of GI manifestations may portend poor overall disease control [2].

## Conclusions

This case describes an unusual SLE flare, in which chronic inflammatory diarrhea was a central aspect. It was also marked by polyarthritis, pleural effusion, and nephritis. Radiological features and exclusion of other causes were fundamental to establishing the diagnosis of LE, which should be carefully considered in patients with SLE who experience persistent GI symptoms. Rapid identification and timely treatment with immunosuppressive therapy can improve clinical outcomes and help prevent bowel ischemia. Furthermore, strict adherence to lupus medications is vital to minimize the risk of flare recurrence. Ongoing monitoring is essential to ensure the sustained resolution of symptoms and stable organ function.
